# Effects of Hyperbilirubinemia on Auditory Brainstem Response of Neonates Treated with Phototherapy

**Published:** 2016-01

**Authors:** Negin Salehi, Fereshte Bagheri, Hamid Ramezani Farkhani

**Affiliations:** 1 *Department of Audiology, School of Rehabilitation, Iran University of Medical Sciences, Tehran, Iran.*

**Keywords:** Auditory Brainstem Response, Hyperbilirubinemia, total serum bilirubin, neonate

## Abstract

**Introduction:**

One of the most common pathologies in neonates is hyperbilirubinemia, which is a good marker for damage to the central nervous system. The sensitivity of the auditory system to bilirubin has been previously documented, with much discrepancy in its effects on Auditory Brainstem Response results. Thus the objective of this study was to evaluate the effects of hyperbilirubinemia on Auditory Brainstem Response of neonates treated with phototherapy.

**Materials and Methods:**

Forty-two term neonates with hyperbilirubinemia, who underwent phototherapy participated in this cross sectional study. The recording of Auditory Brainstem Response was made shortly after confirming that the total serum bilirubin level was greater than 15 µg/dl. Latency of waves I, III, V and inter-peak latencies of the waves were measured. To test the hypothesis about the difference of means between the two groups, continuous variables were compared using either the t-test (normal distribution) or the Mann-Whitney test (non-normal distribution).

**Results:**

There was a significant increase in the absolute latencies of waves III and V, and I-III and I-V inter-peak latencies of the sample group compared to the control group in both ears (P<0.05). However, wave I absolute latency and III-V inter-peak interval did not show a significant difference between the two study groups (P>0.05).

**Conclusion:**

The results of this study underline the importance of the Auditory Brainstem Response Test as an efficient tool for monitoring the auditory brainstem pathway in neonates who are at risk of neurotoxicity and for diagnosing the earliest stages of auditory damage caused by high levels of bilirubin.

## Introduction

One of the most common pathologies in neonates is hyperbilirubinemia, which clinically manifests as jaundice (1,2). Screening of jaundice is suggested in all neonates for early detection and treatment of hyperbilirubinemia (3). Total serum bilirubin (TSB) and neonates’ gestational age are the two most important factors in evaluating risk factors in these screening guidelines, which are that bilirubin's level is interpreted according to the neonate’s age in hours (4,5). 

Generally, hyperbilirubinemia is a good marker for neurologic complaint and increase in bilirubin's level is accompanied by increased likelihood of damage to the central nervous system (CNS) (6). The dysfunction induced by hyperbilirubinemia includes a wide range of neurological damage, dependent on acute or chronic exposure of CNS to bilirubin. This spectrum includes Kernicterus, acute bilirubin encephalopathy, and isolated neural pathway dysfunction (7).

Up to 40 percent of neonates with jaundice are at risk of hearing loss (8). The sensitivity of the auditory system to bilirubin has been previsouly documented and many researchers reported the relationship between hyperbilirubinemia and damage to the auditory system (9-11). Auditory brainstem nucleuses and inferior colliculus are affected by hyperbilirubinemia (12). Additionally abnormalities in spiral ganglion neurons and myelinated auditory fibers are reported (13). Some studies have shown that a baby's auditory system is influenced by neonatal jaundice (14-16).

The damage to the auditory system has long-term and permanent effects, since language development is completely dependent on auditory function (17). Auditory Brainstem Response (ABR) is a reliable and objective electrophysiological method for evaluating ascending auditory systems. It relies on recording the electrical activity of the auditory system that occurs in response to an appropriate acoustic stimulus (18). 

A review of literature reveals that most of the studies have shown abnormal ABR results in infants with hyperbilirubinemia, usually with an increase in the wave's latency. ABR abnormality in these infants is recommended as an indication of bilirubin ototoxicity (19-21). On the other hand, another category of studies did not find any abnormalities in these groups of patients (22-24) .Thus the present study aimed at evaluating the effects of Hyperbilirubinemia on Auditory Brainstem Response of neonates treated with phototherapy.

## Materials and Methods

*Patients*

This cross-sectional study included forty two term neonates (20 boys, 22 girls) who were diagnosed with hyperbilirubinemia within 10 days after birth. The mean total bilirubin ≥15 µg/dl, ranged between 15.4, 27.6 µg/dl (18.7 ± 3.1 µg/dl). The neonates underwent phototherapy in Imam Khomeini hospitals of Tehran, between October and December 2012. Birth weight in the study group ranged between 2700, 4500 g (3255.48±449.11 g).

The exclusion criterion for this study was presence of neonatal hypoxia (assessed as Apgar value lower than 7 in the fifth minute), intrauterine infections, sepsis or meningitis and craniofacial malformations, a family history of hearing loss, low birth weight, ototoxic medication, congenital malformations, and altered otoscopy.

The control group was composed of forty term neonates who did not have any major perinatal conditions and showed no evidence of hyperbilirubinemia. Their birth weight ranged between 2800, 4700g (3555± 350.23g). ABR recordings were made 10 days after the birth. At the time of the test, gestational age ranged between 37 and 40 weeks (38.75 ± 0.55) in the control group and 37, and39 weeks (38.59±0.73 weeks) in the study group, which did not have any significant differences. It should be noted that although ABR is a completely noninvasive technique, written parental consent was obtained prior to the study.

*ABR testing*

The recording of ABR was made within 24 hours after confirming that the TSB level was greater than 15 µg/dl. Prior to ABR recording, the external acoustic canal was inspected to prevent any blockage of the meatus by wax or collapsing the acoustic lumen. Both ears were tested in all neonates. The ABR recordings began after the neonates had had natural sleep. A Charter model Evoked potential system by Denmark was used for ABR recordings. Three disk electrodes were placed at ipsilateral earlobe (negative), contralateral earlobe (positive), and middle forehead (ground). The interelectrode impedance was kept below 5 kΩ during the test. The acoustic stimulus was rarefaction clicks of 100 µs that was presented monaurally to TDH 39 earphones. 2000 stimuli of 80 dB normal hearing level by repetition rate of 21.1 /second was averaged in time window of 15 ms. 2048 brain responses were amplified and filtered by bandpass filer of 100-3000 Hz.

*Data analyses*

Latency of waves I, III, V and inter-peak latencies of the waves were measured and calculated after averaging the two replicable recordings without any knowledge about the neonates medical history. In order to test the hypothesis about the difference of means between the two groups, continuous variables were compared using either the t-test (normal distribution) or the Mann-Whitney test (non-normal distri bution).

## Results

Comparison of ABR absolute latencies (mean±sd) in neonates with hyperbili rubinemia and normal controls are shown in table 1. As can be seen in the table, there is a significant increase in absolute latencies of waves III and V in the sample group compared to the control group in both ears (P<0.05). However, the wave I absolute latency does not show significant difference between two study groups (P>0.05).

**Table1:** Measurements of ABR absolute latencies in neonates with hyperbilirubinemia and normal controls

ABR variables	Ear	Group	Mean ± sd	Statistics	P
I Latency (ms)	R	sample	1.35 ± .11	-1.04	0.29
		Control	1.31 ± .10		
	L	sample	1.33 ± .11	-0.46	0.64
		Control	1.30 ± .09		
III Latency (ms)	R	sample	4.23 ± .26	18.80	<0.0001
		Control	3.40 ± .10		
	L	sample	4.23 ± .24	-7.72	<0.0001
		Control	3.42 ± .23		
V Latency (ms)	R	sample	7.90 ± .70	-7.79	<0.0001
		Control	5.71 ± .23		
	L	sample	7.82 ± .65	-7.76	<0.0001
		Control	5.76 ± .32		

On the other hand, table 2 shows comparisons of ABR interpeak latencies in neonates with hyperbilirubinemia and normal controls. I-III and I-V interpeak latencies are significantly longer in the sample group compared to the control group (P<0.05). There is no significant difference in the III-V interpeak interval between the two study groups (P>0.05).

**Fig1:**
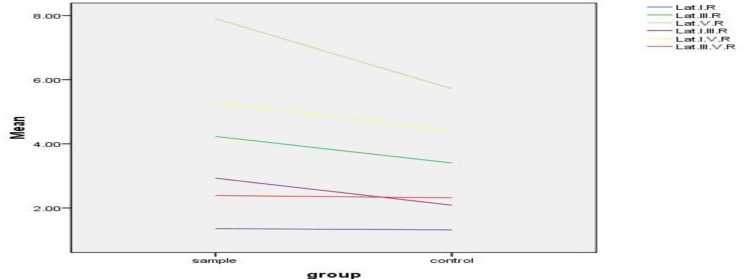
Lat.I.R: Latency of wave I in Right ear

Fig 1 shows the mean of the ABR variable in the right ear of both study groups. It is obvious that wave V has the most difference between these two groups.

**Table 2:** Measurements of ABR inter-peak latencies in neonates with hyperbilirubinemia and normal controls

ABR variables	Ear	Group	Mean ± sd	Statistics	P
I-III Inter-peak (ms)	R	sample	2.92 ± .23	19.44	<0.0001
		Control	2.08 ± .14		
	L	sample	2.94 ± .24	-7.76	<0.0001
		Control	2.12 ± .25		
I-V Inter-peak (ms)	R	sample	5.29 ± .36	13.18	<0.0001
		Control	4.40 ± .23		
	L	sample	5.35 ± .33	-7.46	<0.0001
		Control	4.45 ± .34		
III-V Inter-peak (ms)	R	sample	2.38 ± .21	-0.60	0.54
		Control	2.31 ± .27		
	L	sample	2.42 ± .21	-1.36	0.17
		Control	2.33 ± .34		
					

Lat.III.R:Latency of wave III in Right ear

Lat.V.R:Latency of wave V in Right earLat.I-III.R: Interpeak Latency of I-III in Right ear

Lat.I-V.R:Interpeak Latency of I-V in Right ear

Lat.III.V.R:Interpeak Latency of III-V in Right ear

## Discussion

Significant increase in unconjugated bilirubin in neonates, with other peripheral risk factors, makes bilirubin cross the blood brain barrier and induce acute encephalopathy. In neonates with severe and long term hyperbilirubinemia, deposition of unconjugated and free bilirubin in special brain areas causes encephalopathy (25,26). In spite of large developments in medicine in recent years, hyperbili rubinemia and its effects on sensory and motor systems is still a major problem (27). Early discharge of neonates from the hospital, inadequate knowledge about severe effects of hyperbilirubinemia, and incomplete follow-up programs in these high risk group are mentioned as the main causes of this big problem in previous studies (28,29). 

The neonatal auditory system is very sensitive to high levels of bilirubin and can be affected in hyperbilirubinemia (30). 

It is well known that neonatal ABRs have three waves, I, III and V with latency values that have physiological and clinical importance, because change in latency values is indicative of disturbances in the auditory brainstem function (31).

Wave I originates from spiral ganglion cells of the auditory nerve that connect to the cochlea. Waves III and V are attributed to lower and upper brainstem areas, respectively (32). 

In our study, like most of the previous studies in this context (33-35), absolute latencies of waves III and V are significantly longer in neonates with hyperbilirubinemia than normal controls. As there was not any increase in wave I latency in these neonates, the increase in later waves may be due to an abnormality in the central auditory pathway. This is confirmed by longer I-III and I-V interpeak intervals that show brainstem conduction time.

Biochemical and physiological evidences introduce the synapses as the primary target for bilirubin effects. Synapses along the auditory brainstem pathway can be disturbed severely (26). This is supported by an increase in I-III and I-V interpeak latencies, which are found in this study. 

As it is demonstrated in Figure 1, most of the increase occurred in wave V absolute latency and consequently the increase in I-V interpeak latency is greater than a similar case in the I-III interpeak latency. This finding shows that the rostral regions of the brainstem, rather than the caudal ones, are more sensitive to an increase in bilirubin levels.

There was not any significant difference in wave I absolute latency between both study groups in this study. This is in agreement with previous studies and can be due to the non-involvement of the cochlear nerve (16,36). If the hyperbilirubinemia is very severe, an increase in wave I absolute latency can be observed as well (37,38). 

The results of this study and previous studies demonstrate that auditory brainstem nucleuses are the main target of bilirubin effects in these neonates. Thus using the Oto-acoustic Emission (OAE) test alone in screening programs for high risk neonates, before discharge from the hospital, is obviously inadequate (11,38,39). On the other hand, these central auditory impairments, which are observed in this study, can have important clinical implications. We did not follow-up the neonates after treatment or exchange transfusion. But persistence of ABR abnormalities in some cases, even after discharge from the hospital (shown in previous studies), can indicate axonal degeneration and loss of myelin and highlight the importance of rapid treatment (40). Thus differentiation of peripheral from central auditory impairment and special attention to central impairments in these patients after neonatal hyperbilirubinemia has great importance. It must be noted that as the neonates in this study did not have any pathological process that could have affected ABR responses, the observed changes in latency values can be mainly attributed to an increase in bilirubin levels.

## Conclusion

Results of this study underline the importance of auditory evoked potentials in evaluating the neonatal auditory system. ABRs can be an efficient tool for monitoring the auditory brainstem pathway in neonates who are at risk of neurotoxicity. Diagnosing the earliest stages of auditory damage caused by high levels of bilirubin is key at a stage where lasting central effects may be preventable. 
